# *Bactrocera dorsalis* male sterilization by targeted RNA interference of spermatogenesis: empowering sterile insect technique programs

**DOI:** 10.1038/srep35750

**Published:** 2016-10-21

**Authors:** Yong-Cheng Dong, Zhi-Jian Wang, Zhen-Zhong Chen, Anthony R. Clarke, Chang-Ying Niu

**Affiliations:** 1 Hubei Insect Resources Utilization and Sustainable Pest Management Key Laboratory, College of Plant Science & Technology, Huazhong Agricultural University, Wuhan, 430070, China; 2 School of Earth, Environmental and Biological Sciences, Faculty of Science and Technology, Queensland University of Technology (QUT), P.O. Box 2434, Brisbane QLD 4001, Australia

## Abstract

RNA interference (RNAi) is a genetic technique which has novel application for sustainable pest control. The Sterile Insect Technique (SIT) uses releases of mass-produced, sterile male insects to out-compete wild males for mates to reduce pest populations. RNAi sterilization of SIT males would have several advantages over radiation sterilization, but to achieve this appropriate target genes must first be identified and then targeted with interference technology. With this goal, eight spermatogenesis related candidate genes were cloned and tested for potential activity in *Bactrocera dorsalis*. The knockdown of candidate genes by oral delivery of dsRNAs did not influence the mating of male flies, but significantly affected the daily average number of eggs laid by females, and reduced egg hatching rate by 16–60%. RNAi negatively affected spermatozoa quantitatively and qualitatively. Following the mating of *lola*-/*topi*-/*rac*-/*rho*-/*upd*-/*magu*-silenced males, we recorded a significant decrease in number and length of spermatozoa in female spermatheca compared to *gfp*-silenced control group. In a greenhouse trial, the number of damaged oranges and *B. dorsalis* larvae were significantly reduced in a ds*rho*-treated group compared with the ds*gfp* group. This study provides strong evidence for the use RNAi in pest management, especially for the improvement of SIT against *B. dorsalis* and other species.

The RNA interference (RNAi) phenomenon is a conserved biological defense response which mediates resistance to both endogenous, parasitic, and exogenous pathogenic nucleic acids in a sequence-specific manner^1^. In eukaryotes, RNAi involves exposure to double-stranded RNA (dsRNA) molecules resulting in post-transcriptional degradation of homologous messenger RNA (mRNA) causing corresponding loss-of-function^2^. Gene silencing through the RNAi technique has been recognized as a powerful research tool in genomics, medicine and biotechnology^3^,^4^, as well as being a promising technology outside the laboratory for the applied biological sciences in fields such as agricultural pest management^5^,^6^. RNAi invoked gene silencing can be promoted by either direct feeding of dsRNA to an organism, or by engineering plants or bacteria to produce dsRNA^3^: both approaches are operationally feasible for basic research and practical application^6^,^7^,^8^,^9^,^10^. However, regardless of its eventual use, implementing sequence-specific RNAi approaches always requires the screening of target genes, which in insect pest management (as one example) includes detoxifying enzymes^8^ and chitin synthase genes^11^.

The sterile insect technique (SIT) is an environmentally friendly insect pest management technique which operates by disrupting reproduction. Males of the target species are mass-reared in factories, sterilized, and then released. The sterile males mate with wild females, making their eggs sterile in turn and causing the wild population to crash^12^. Many of world’s major agricultural and human health pests are amenable and targeted for SIT control, including mosquitoes, screwworms, tsetse flies and tephritid fruit flies^13^. To date, sterilization of factory-reared flies is almost always achieved through ionizing radiation which, while effective, is limiting because of the need for an appropriate radiation source and the unavoidable loss of competitive ability in treated male flies due to somatic damage^14^,^15^. These detrimental effects have prompted studies to find alternative sterilization strategies. Sequence-targeted RNAi is one promising approach to replace irradiation sterilization by pinpointing the genes responsible for spermatogenesis, and the circumvention of somatic damage.

Spermatogenesis encompasses seven distinct differentiation stages: these are the formation of hub cells, cyst stem cells, cyst cells, germline stem cells (GSCs), spermatogonia, spermatocytes and spermatids. These stages are regulated by dynamic gene expression changes at transcriptional, post-transcriptional and post-translational levels^16^. Several key signaling pathways which exert transcriptional regulatory function on spermatogenesis include Bone Morphogenetic Protein (BMP) signaling^17^, Janus Kinase-Signal Transducer and Activator of Transcription (JAK-STAT) signaling^18^, and Egf and Egfr signaling^19^. BMP and JAK-STAT pathways execute the roles of GSCs maintenance^17^, while the *magu* gene can modulate BMP signaling to control GSCs maintenance in the testis niche^20^, and the transcriptional regulator *longitudinals lacking* (*lola*) is required for stem cell maintenance^21^.

In addition to *magu* and *lola* mentioned above, *unpaired* (*upd*) gene is the ligand of the JAK-STAT signaling pathway^19^. *Rac* and *rho* are gene products downstream of the Egf pathway expressed in cyst stem cells and cyst cells stages^16^: belonging to the members of Rho GTPases they regulate various cellular functions, especially spermatogenesis^22^,^23^. *Always early* (*Aly*) and *Matotopetli* (*Topi*) belong to *aly*-class meiotic arrest genes and are involved in the processes of spermatid differentiation^24^,^25^,^26^,^27^. In addition, circadian clock genes not only govern insect daily rhythms that strongly influence an insect’s reproductive behavior, *e.g.*, the *period* (*per*) which affects sperm release^28^,^29^, but these genes also affect the normal progress of spermatogenesis and oogenesis^30^. While all these genes offer targets of potential use for sterilization in SIT pest management, nearly all RNAi experiments have been carried out at the cellular level^31^,^32^,^33^ and our knowledge is limited on whether the approach could be efficiently applied for mass production of sterile males as required for SIT projects.

Our insect model of interest is the Oriental fruit fly, *Bactrocera dorsalis* (Hendel) (Diptera: Tephritidae), a devastating agricultural pest which attacks a broad range of fruits and vegetables^34^, in tropical and subtropical zones from Africa, across Asia (including China) and into the Pacific. Previous molecular studies on this pest have revealed that an oral application of dsRNA modifies their gene expression patterns^35^,^36^, identifying this fly as a suitable candidate for further research. As spermatogenesis candidate genes are potentially appropriate targets for RNAi male sterilization, the selection of these genes will be a key factor towards the eventual application of this approach^37^. Our objectives were therefore to identify suitable candidate genes, and then test whether an orally administered engineered-bacteria expressing spermatogenesis related dsRNAs can lead to male sterility and reduce pest impacts in a semi-natural environment. Our results identify appropriate target genes for RNAi sterilization of male *B. dorsalis* and a reduction of fruit damage following oral feeding. This illustrates the promise of RNAi technology as an alternative method to irradiation in SIT programs and its future value in the integrated pest management of *B. dorsalis* and other agricultural pests.

## Results

### Cloning of target genes

Based on our transcriptomic data, RT-PCR was used to amplify the partial sequence of the target genes (Table 1). The fragments of *topi*, *per*, *aly*, *rac* and *magu* encompassing complete coding sequence (CDS) of a 1092, 3135, 2001, 579 and 1602 bp open reading frame (ORF), encoded 363, 1044, 666, 192 and 533 amino acids respectively, which were highly conserved showing high percentages of identity to these genes in other *Bactrocera* species. Partial cDNA sequence of *lola*, *rho* and *upd* were isolated, encoding 485, 272 and 220 amino acids respectively, which shared a high homology in sequence alignment with *Bactrocera oleae* (Rossi).

### Effects of RNAi on *B. dorsalis* reproduction

**Figure 1:**
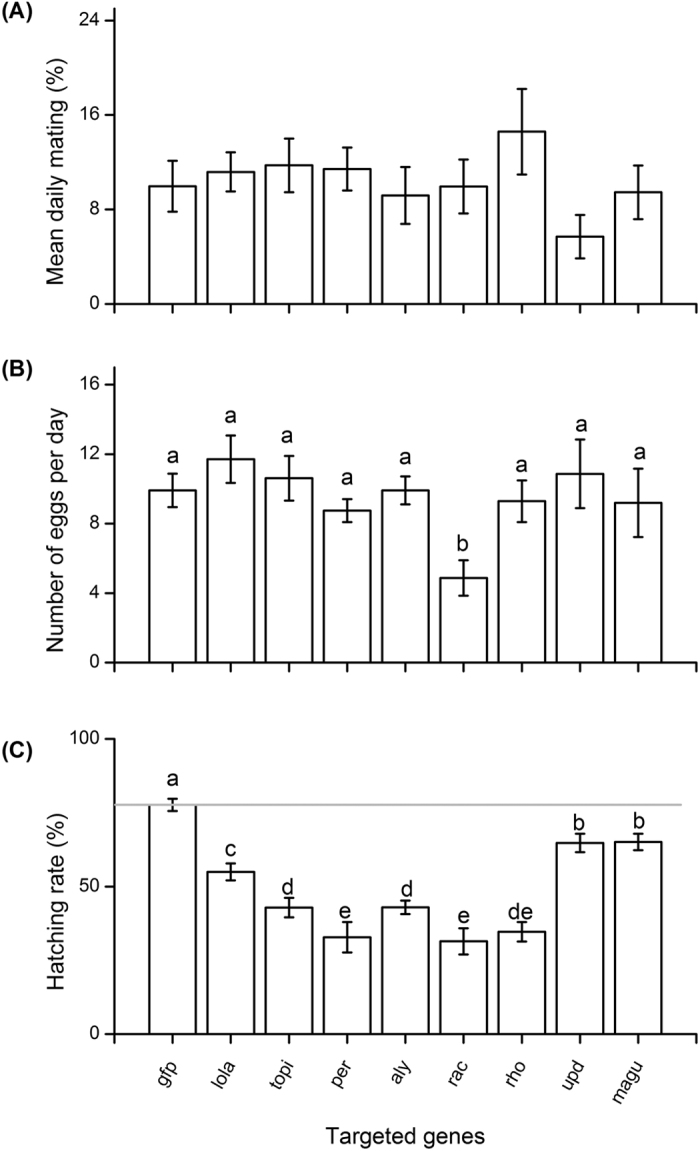
The mean (**A**) proportion of valid matings per day, (**B**) number of eggs laid per day and (**C**) the egg hatching rate of *Bactrocera dorsalis* among treatment groups after oral delivery of bacteria expressing different spermatogenesis related dsRNAs. The green fluorescent protein double-stranded RNA (ds*gfp*) treatment group was used as a control. All the experiments were performed in triplicate. Histograms represent mean ± SE values and different letters indicate significant differences among groups at <0.05 level (ANOVA followed by Fisher’s Least Significant Difference (LSD) *post-hoc* test).

Different dsRNA treatments did not influence male mating ability, as there was no significant difference for the percentage of valid matings between dsRNA treated and negative control groups (*F*_*8,126*_ = 1.062, *P* = 0.394, Fig. 1A). The daily number of eggs laid by females did differ significantly after mating with dsRNA-treated males (*F*_*8,126*_ = 2.179, *P* = 0.033), but *post hoc* tests identified only the ds*rac* gene treatment group differed significantly from all other treatments (Fig. 1B). Egg hatching rate was significantly (*F*_*8,126*_ = 24.142, *P* < 0.001) reduced for all target genes from 16% (ds*magu*) to 60% (ds*rac*) (Fig. 1C) compared with the ds*gfp* treated control group.

### Effects of RNAi on target gene expressions

**Figure 2:**
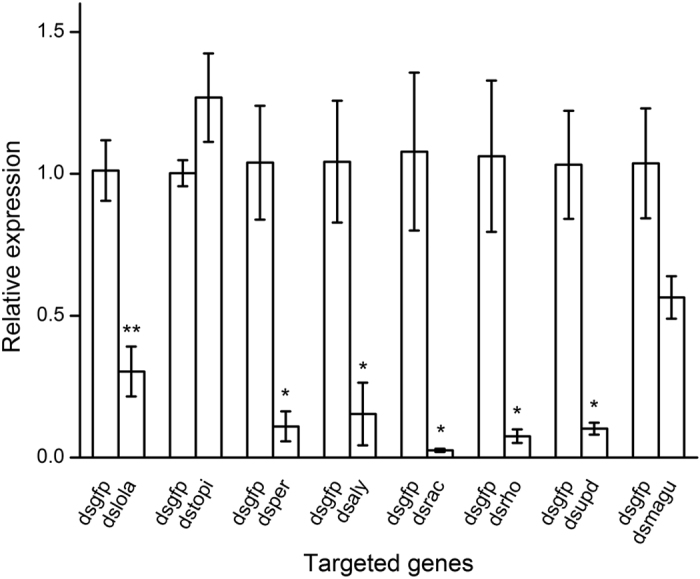
The mean (±SE) relative gene expression of *Bactrocera dorsalis* male spermatogenesis related genes after oral administration of bacteria expressing dsRNAs of target genes. The green fluorescent protein double-stranded RNA (ds*gfp*) treatment group was used as a negative control. All the experiments were performed in triplicate. Asterisks indicate significant differences between ds*gfp* and ds*RNA*s groups (Independent sample *t* test, *P < 0.05; **P < 0.01).

To evaluate the silencing effects of target dsRNA treatments, *B. dorsalis* target gene expressions were detected by qPCR. Results showed that the expression levels of *lola* (*t* = 5.130, *P* = 0.007), *per* (*t* = 4.480, *P* = 0.011), *aly* (*t* = 3.688, *P* = 0.021), *rac* (*t* = 3.782, *P* = 0.019), *rho* (*t* = 3.683, *P* = 0.021) and *upd* (*t* = 4.854, *P* = 0.038) from the male adults of corresponding feeding groups were significantly decreased compared to the control (ds*gfp*) group. Expression levels of *magu* (*t* = 2.276, *P* = 0.085) and *topi* (*t* = −1.642, *P* = 0.224) were not statistically different from the control (Fig. 2).

### Effects of RNAi on sperm quantity and quality in spermatheca of females

**Figure 3:**
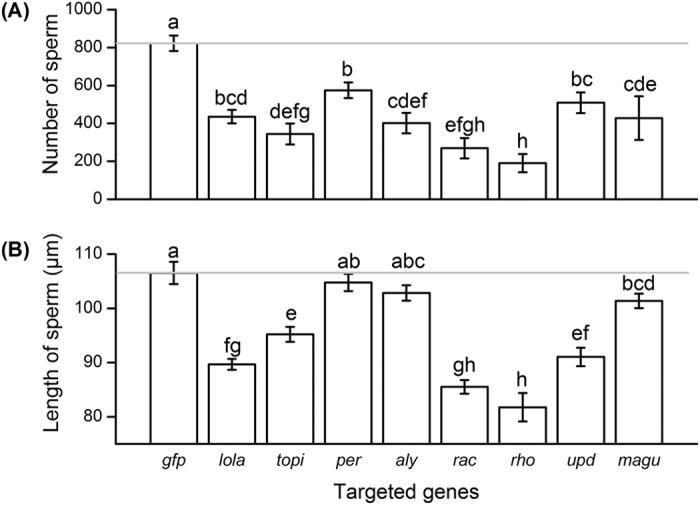
The mean (±SE) (**A**) number and (**B**) length of sperm in the spermatheca of female flies after mating with male *Bactrocera dorsalis* having received orally administered RNA interference treatments on male spermatogenesis related genes. The green fluorescent protein double-stranded RNA (ds*gfp*) treatment group was used as a negative control. Different letters above columns indicate significant differences among groups at <0.05 level (ANOVA followed by Fisher’s Least Significant Difference (LSD) post-hoc test).

RNAi targeting spermatogenesis related genes significantly influenced the spermatozoa stored in spermatheca of female flies both in quantity (sperm number: *F*_*8,107*_ = 11.111, *P* < 0.001) (Fig. 3A) and in quality (sperm length: *F*_*8,172*_ = 29.357, *P* < 0.001) (Fig. 3B). The number of spermatozoa in spermatheca were significantly reduced in all treatments compared with ds*gfp* treated group, with the reduction ranging from 30% (ds*per*) to 77% (ds*rho*) (Fig. 3A). ds*per* and ds*aly* treatments did not significantly reduce sperm length compared to the ds*gfp* control group, but the other gene treatment groups had shorter sperm than the control treatment, reduced by 5% in the ds*magu* group to 20% in the ds*rac* group (Fig. 3B). Although not quantified, other morphological changes were also observed to occur in the shape of sperm. The anterior of sperm appeared abnormally enlarged in the ds*rho* treated group (Fig. S1).

### Greenhouse trials

**Figure 4:**
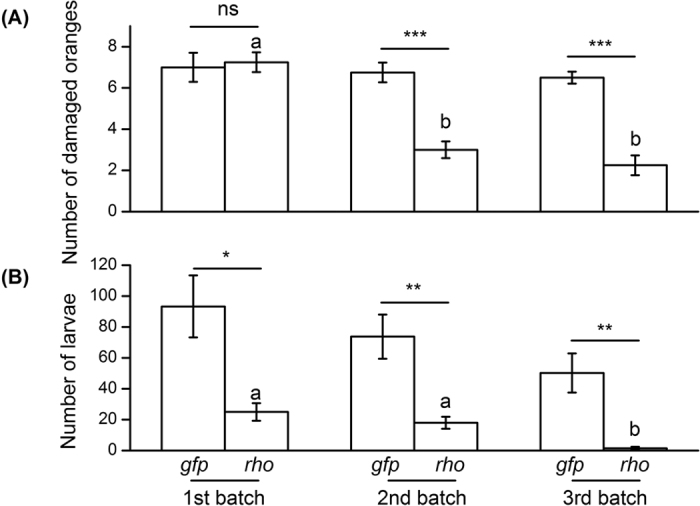
The mean (±SE) number of (**A**) damaged oranges and (**B**) *B. dorsalis* larvae in those oranges from ds*gfp* and ds*rho* oral administration groups in a greenhouse cage trial. Mean values were compared using two-way ANOVA, followed by Fisher’s Least Significant Difference (LSD) *post-hoc* test. Different letters indicate the significant differences among different batches of oranges, while asterisk indicates the significant difference between ds*gfp* (control) and ds*rho* treatments (*P < 0.05, **P < 0.01; ***P < 0.001).

ds*rho* treatment in the greenhouse cage trials significantly diminished the number of damaged oranges and the total larval number (Table 2, Fig. 4). There was no significant difference for the damage proportion between ds*rho* and ds*gfp* group for the 1^st^ batch of fruits, but the differences became noticeable and significant for the 2^nd^ and 3^rd^ batches (Fig. 4A). There were significant differences for total larval number between ds*rho* and ds*gfp* group for all the three batches of fruits (Fig. 4B). No significant difference was observed for both damage rate and larval number across three batches of fruits within ds*gfp* groups. However, both the numbers of damaged oranges and *B. dorsalis* larvae across three batches of fruits within ds*rho* group significantly decreased in a stepwise manner (Fig. 4). Feeding ds*rho* in the greenhouse trial significantly reduced the transcriptional level of *rho* by 93% (*t* = 5.694, *P* = 0.025), which was identical to the gene silencing effect in the corresponding laboratory experiment (decrease of *rho* by 93%, Fig. 2).

## Discussion

Eight spermatogenesis related genes in *B. dorsalis*, namely *lola*, *topi*, *per*, *aly*, *rac*, *rho*, *upd* and *magu*, were cloned and their potentials in pest control application were evaluated by orally supplied engineered-bacteria expressing target dsRNA. The results showed that silencing *lola*, *topi*, *rac*, *rho*, *upd* and *magu* severely impaired male sperm in both quality and quantity, and, in turn, significantly decreased female fertility. Furthermore, the greenhouse cage trials proved successful for controlling *B. dorsalis* by feeding with bacteria expressing ds*rho*, which resulted in less damaged oranges and less larvae. Combining our laboratory and field-cage experiments, our results provide well-chosen target genes related to spermatogenesis and reveal a significant potential for male sterilization in pest management for *B. dorsalis*.

The feeding experiments of RNAi to *B. dorsalis* in the present and previous studies^35^,^36^ suggest that oral administration of engineered-bacteria can efficiently suppress the target gene expression, which increases the potential of RNAi technology in pest management^3^,^4^. However, compared with microinjection or other methods of applying RNAi to insects, the silencing efficiency of RNAi by oral administration has sometimes been insufficient due to factors such as the variable concentration of dsRNA delivered, selection of nucleotide sequence, length of dsRNA fragment, and intestinal environment conditions of the target organism^3^,^38^. In the present study, nonetheless, all the target genes significantly responded to the treatments of RNAi except for *magu* which was non-significantly reduced, and *topi* that was non-significantly but, unexpectedly, up-regulated. These non-significant effects may be due to an insufficient concentration of dsRNA received^39^, or some other unknown mechanism such as the development of refractoriness to RNAi. In a trial of RNAi in *Locusta migratoria* (L.) by dsRNA oral delivery, doses ranging from 0.1 μg to 12 μg did not significantly influence the relative expression of V-ATPase E, whereas at 18 μg, an efficient inhibition of mRNA expression was observed^40^. Paradoxically, an up-regulation of target genes following RNAi treatments have been observed in other insects^35^,^41^,^42^, and is thought to be due to involved immunogenic factors^43^. Ultimately, appropriate and accurate sequence selection from the target gene is one of the most important factors influencing a successful outcome of the silencing effect^39^.

All the orally administered dsRNAs treatments in *B. dorsalis* reduced the number and generally the length of spermatozoa stored in female spermatheca, and significantly reduced offspring hatching rate compared to the control ds*gfp* treatment (Fig. 1C). Although the molecular mechanisms involved are still unclear, it is evident that disrupting the spermatogenesis related genes influenced the morphological characters of spermatozoa *i.e.*, shorter and abnormally head-swollen sperm. This, in turn, might change the driving speed and numbers of spermatozoa in the female spermatheca, leading to subsequent decrease of fertility. Sperm with longer flagellum and shorter heads relative to their flagellum swam faster in externally fertilizing species, but slower in internally fertilizing species^44^. However, in the dung fly, sperm length variation had no association with sperm competitiveness^45^. Further studies are therefore recommended to unravel how the silencing of our target genes affect the mechanics of fertilization in *B. dorsalis*.

The oral delivery of engineered bacteria expressing ds*rho* to adult flies in 0.7 m^3^ cages, which decreased both the number of infested oranges and number of larvae per infested fruit, throw light on RNAi’s practical potential for controlling *B. dorsalis*. At the first harvest the number of damaged oranges was not significantly different from the control group; this might be due to egg “dumping” in the newly available host fruit and high age-specific reproductive ability that peaks in an early oviposition period in anautogenous tephritids^46^ such as *B. dorsalis*. However, over the course of the experiment, the total reproductive ability of *B. dorsalis* was suppressed in the ds*rho* treated group with the efficacy of pest control strengthened in a stepwise fashion owing to the persistently reinforced RNA silencing effects. Repetitive oral delivery of dsRNA may be comparable to multiple microinjection, where a double injection was more effective than a single injection of dsRNA in fourth-instar nymphs of *Rhodnius prolixus* Stål^47^. The greenhouse cage results indirectly suggested deleterious effects of ds*rho* on females, since the decrease in the percentage of infested oranges over time hints that oviposition activities were inhibited. In *Drosophila*, *rho* was necessary for the development of the dorsal-ventral axis in oogenesis, and loss of *rho* function induced ventralization of the eggshel^48^. Thus, *rho* may exert inhibitory actions on both spermatogenesis and oogenesis in *B. dorsalis*, but additional work is needed to confirm or deny this hypothesis.

RNA interference is a powerful molecular method with significant potential for sustainable pest control. Compared to radiation sterilization methods used in SIT programs and that subsequent indirect fitness costs to males, gene-specific silencing technology represents a novel and environmental-friendly male sterilization approach which not only circumvents the need for a radiation source, but also directly generates the target function loss *i.e.*, reproductive capacity, without other forms of adult fitness cost. Our work demonstrates that the *rho* gene is the most potent target for *B. dorsalis* pest control. Orally administered, the target dsRNAs in laboratory and large cage conditions effectively silenced the target genes’ expression and lowered subsequent reproduction capacity, thereby demonstrating the technology’s potential for controlling *B. dorsalis*. However, in order for this approach to be commercially used, many challenges need to be overcome such as bringing the sterilization success rate to nearly 100%, confirming species-specific gene selection, testing off-target effects and determining how dsRNA could be implemented into the SIT approach, for example as a pre-release or post-release treatment.

## Materials and Methods

### Insects

A near-wild laboratory colony of *B. dorsalis* was originally field-collected from a citrus orchard in Wuhan city, Hubei Province, China, and was reared for two to three generations before used for the experiments. Adults were held in 40 cm × 30 cm × 30 cm cages and had free access to a liquid artificial diet (200 g sugar, 60 g tryptone, 40 g brewers’ yeast, 1 L distilled water). Adults were egged using an artificial egging device (a pinpricked yellow plastic cup on 3 cm petri dish spotted with orange juice to collect eggs) and larvae were raised on artificial mill feed diet^49^. All life stages were held at 26 ± 1 °C, RH 70 ± 5% and a photoperiod cycle of 14 L: 10 D.

### Cloning of target genes

Adult males were sampled daily from day 1 to 30 after emergence (DAE). Total RNA was isolated with TRIzol reagent (Invitrogen) following the manufacturer’s instructions. Total RNA was incubated with 10 U DNase I (Thermo Scientific, USA) at 37 °C for 30 min for mRNA purification. First strand cDNA was produced from 5 μg RNA using Revert Aid First Strand cDNA Synthesis Kit (Thermo Scientific, USA). Target gene sequences (*lola*, *topi*, *per*, *aly*, *rac*, *rho*, *upd*, *magu*) were obtained by retrieving previously constructed *B. dorsalis* transcriptome data (Y.-C. D., Z.-J. W., C.-Y. N., unpublished data), and their gene specific primers were designed using Primer Premier 5.0 (Premier, Canada) (Table S1). PCR amplicons were purified using AxyPrep DNA Gel Extraction Kit (AxyPrep, USA). The purified products were ligated to cloning vector by using pMD™ 18-T Vector Cloning Kit (TaKaRa, China). The plasmid recombinants (pMD-18T-*lola*, -*topi*, -*per*, -*aly*, -*rac*, -*rho*, -*upd*, -*magu*) were amplified by PCR and verified by Sanger sequencing (Invitrogen, Shanghai, China).

### Expression of dsRNA

The L4440 plasmid, comprising two T7 promoters in inverted orientation flanking the multiple cloning site, was applied for target dsRNA inducible expression. Double restriction enzyme digestion was used to cut pMD-18T-*genes* and L4440 plasmid, respectively. The restriction enzyme digestion sites were checked by using Primer Premier 5.0 to ensure their presence exclusively in pMD-18T and L4440 plasmid, other than in that of the target genes (Table S2). The target fragments were excised from pMD-18T-*genes* and ligated to L4440 plasmid by T4 DNA Ligase (TaKaRa, China). The recombinant vectors (L4440-*lola*, -*topi*, -*per*, -*aly*, -*rac*, -*rho*, -*upd*, -*magu*) were transformed to *Escherichia coli* HT115 competent cells which lack RNase III (Competent Cell Preparation Kit, TaKaRa, China). Single colonies of HT115 were cultured in Luria Broth (LB) media at 37 °C with shaking at 220 rpm overnight. The culture was diluted 100-fold in 2 L LB with 100 μg/ml ampicillin cultured at 37 °C and 0.6 optical density 600. Synthesis of T7 polymerase was induced with 0.4 mM IPTG and the bacteria were incubated with shaking for an additional 4 h at 37 °C. Bacteria solutions of HT115 were centrifuged at 4,000 g for 5 min and re-suspended in 4 ml distilled water to condense the concentration to 500×.

### Feeding target dsRNA

Newly-emerged males and females were separated into individual rearing cages with each cage containing ~200 males or females. A 500 μl liquid artificial diet + 1,500 μl 500× bacteria expressing target dsRNA, was applied on a filter paper spotted in a petri dish (d = 3.5 cm) to feed male flies, whilst 2,000 μl of the same diet was supplied for females. The negative control group was fed with 500 μl liquid artificial diet + 1,500 μl 500× bacteria expressing ds*gfp*. Liquid diet was daily renewed at 9:00 am from DAE 1 to 14. The experiment was performed in triplicate. At DAE 12, three males fed with dsRNA were randomly collected for qPCR analysis. By day 14, *B. dorsalis* under normal laboratory conditions would be sexually mature and ready to mate^50^.

### Reproduction bioassays

Having been fed with dsRNA for 14 days, 30 pairs of virgin males and females were put together in a fresh cage. Pinpricked yellow plastic cups were used to collect eggs from 14:00 to 22:00 hrs. Eggs were then removed from cups and placed onto a moist, black filter paper (MACHEREY-MAGEL, Germany) for counting and assessing hatching rate. The numbers of ‘valid matings’ (herein defined as a copulation which lasted for more than one hour) were counted during the mating period^51^, from 19:00 to 21:00 hrs. Both the numbers of eggs and valid mating pairs were recorded every other day from DAE 14 to 24. The experiment was replicated three times.

### Target genes quantitative real-time PCR (qPCR)

qPCR was performed using SYBR Premix Dimer Eraser (TaKaRa) according to the manufacturer’s instructions on ABI 7300 (Applied Biosystems, USA). qPCR primers were designed by Primer Premier 5.0 (Table S3). *Gapdh* (GenBank: GU269901.1) was chosen as an internal control gene. The assays were performed in triplicate on the flies sampled at DAE 12. The relative gene expression data were calculated using 2^−ΔΔ*C*T^ method as described by Livak and Schmittgen^52^.

### Sperm observation

After valid mating, slides for microscopic examination of sperm stored in the spermatheca were prepared immediately using the methods described by Taylor *et al.*^53^ and Aaron *et al.*^54^. Under a stereomicroscope, each spermatheca was transferred individually by clasping its duct with fine forceps to a new slide in deionized water. The spermatheca was broken apart using an entomological pin. The contents of the spermatheca were stirred vigorously in a water drop for 1 min and spread to a diameter of about 15 mm before being covered by an 18 × 18 mm cover slip. The cover slip was secured with nail polish on each corner. After drying overnight, the number and length of sperm from each spermatheca were recorded and the shape was observed at 400× under a phase contrast microscope. Twenty females were dissected for each group, drawn from across the three replicate cages.

### Greenhouse trials

To verify our laboratory results and test the future potential of RNAi, the most efficient sterilizing gene from laboratory studies (*rho*, see Results) was selected for a greenhouse cage experiment by comparing the proportion of damaged citrus fruits and offspring larval number between ds*rho* and ds*gfp* (control) treated groups. Twenty pairs of newly emerged adults were released into a mesh cage (1 m × 1 m × 0.7 m), which contained one citrus plant (about 90 cm tall) without fruit. The cages were kept in open-walled glass-houses, the roof of which provided protection from rain but otherwise the flies in their mesh-cages were exposed to close to ambient field conditions.

A mixed liquid diet of 500 μl artificial diet + 1,500 μl 500× bacteria expressing ds*rho* was supplied for adults in a petri dish with filter paper, whilst the control group was fed with bacteria expressing ds*gfp*. Diet was supplied from DAE 1 and added twice per day (9:00 am and 2:00 pm), throughout the experimental period. At DAE 10, when the flies reached the sexual maturity and were ready to oviposit as recorded in our preliminary work, eight oranges were hung on the branches of the citrus tree and replaced at DAE 15 and 20, with the final harvest at DAE 24. These three batches of eight exposed oranges were then maintained at room temperature (26 ± 1 °C) for five days to allow larval growth, after which they were dissected to count the number of fruits with larval damage and to count the total number of larvae. This cage experiment was replicated four times for each of the two treatments. At DAE 12, three males were collected to detect the relative expression of *rho* in ds*gfp* and ds*rho* treatment groups by qPCR.

### Data analyses

After oral supply of bacteria expressing target dsRNAs for male flies, the results including the number of eggs laid per day, the proportion of valid matings per day (the number of valid mating pairs relative to total pairs of flies), the egg hatching rate, the number and length of spermatozoa in spermathecae of female flies, were analyzed by using one-way analysis of variance (ANOVA) and Fisher’s Least Significant Difference (LSD) *post-hoc* test for multiple mean comparisons. The number of damaged oranges and total larval number for each observation time were analyzed by two-way analysis of variance followed by LSD *post-hoc* test for multiple mean comparisons. Gene expression data in qPCR analysis was compared using independent-samples *t* test. Arcsine square root transformation was applied to any percentage data before statistical analyses. Datasets were tested for normality and homogeneity of variance using Kolmogorov-Smirnov test and Levene’s test respectively, and transformed if needed.

## Additional Information

**How to cite this article**: Dong, Y.-C. *et al.* *Bactrocera dorsalis* male sterilization by targeted RNA interference of spermatogenesis: empowering sterile insect technique programs. *Sci. Rep.* **6**, 35750; doi: 10.1038/srep35750 (2016).

## Figures and Tables

**Table 1:** Eight spermatogenesis related genes cloned in *Bactrocera dorsalis.*

Gene symbol	Length (bp)	Accession	E value	Species
*lola*	1,743	XM_014237796.1	0	*Bactrocera oleae*
*topi*	1,193	XM_014243630.1	0	*Bactrocera oleae*
*per*	3,509	AF480839.1	0	*Bactrocera neohumeralis*
*aly*	2,150	XM_014246922.1	0	*Bactrocera oleae*
*rac*	901	XM_014242198.1	0	*Bactrocera oleae*
*rho*	818	XM_014236354.1	0	*Bactrocera oleae*
*upd*	663	XM_014232453.1	0	*Bactrocera oleae*
*magu*	1,926	XM_011192887.1	0	*Bactrocera cucurbitae*

**Table 2:** Two-way analysis of variance for the proportion of damaged oranges and larval number of *Bactrocera dorsalis* after ds*gfp* and ds*rho* oral administration in the greenhouse cage experiments.

Source of variation	*df*	Number of damaged oranges		Number of larvae	
		*F*	*P* value	*F*	*P* value
*Sampling batch*	2	17.913	**<0.001**	4.127	**0.033**
*Treatment*	1	41.783	**<0.001**	36.634	**<0.001**
*Sampling* × *Treatment*	2	12.696	**<0.001**	0.359	0.703
